# The Right Treatment Strategy for the Right Patient: A Biomarker-Driven Approach to Neoadjuvant vs. Surgery-First Management of Resectable and Borderline Resectable Pancreatic Cancer

**DOI:** 10.3390/cancers14153620

**Published:** 2022-07-25

**Authors:** Christopher B. Nahm, John Turchini, Sumit Sahni, Elizabeth Moon, Malinda Itchins, Jennifer Arena, Angela Chou, Emily K. Colvin, Viive M. Howell, Nick Pavlakis, Stephen Clarke, Jaswinder S. Samra, Anthony J. Gill, Anubhav Mittal

**Affiliations:** 1 Northern Clinical School, Faculty of Medicine and Health Sciences, The University of Sydney, Sydney, NSW 2565, Australia; chris.nahm@sydney.edu.au (C.B.N.); jtur3651@sydney.edu.au (J.T.); sumit.sahni@sydney.edu.au (S.S.); e.millar@sydney.edu.au (E.M.); malinda.itchins@sydney.edu.au (M.I.); angelashihyuan.chou@health.nsw.gov.au (A.C.); emily.colvin@sydney.edu.au (E.K.C.); viive.howell@sydney.edu.au (V.M.H.); nick.pavlakis@sydney.edu.au (N.P.); stephen.clarke@sydney.edu.au (S.C.); jas.samra@bigpond.com (J.S.S.); anthony.gill@health.nsw.gov.au (A.J.G.); 2 Upper Gastrointestinal Surgical Unit, Royal North Shore Hospital, St. Leonards, NSW 2565, Australia; 3 Bill Walsh Translational Cancer Research Laboratory, Kolling Institute, Sydney, NSW 2565, Australia; 4 Australian Pancreatic Centre, Sydney, NSW 2565, Australia; jennifer.arena@genesiscare.com; 5 Westmead Clinical School, Faculty of Medicine and Health Sciences, The University of Sydney, Sydney, NSW 2565, Australia; 6 Surgical Innovations Unit, Westmead Hospital, Hawkesbury Road, Westmead, NSW 2145, Australia; 7 Cancer Diagnosis and Pathology Group, Kolling Institute, Sydney, NSW 2565, Australia; 8 Department of Medical Oncology, Royal North Shore Hospital, St. Leonards, NSW 2565, Australia; 9 Australian Pancreatic Genome Initiative, The Kinghorn Cancer Centre, Garvan Institute of Medical Research, Darlinghurst, NSW 2010, Australia; 10 Faculty of Medical and Health Sciences, Macquarie University, Sydney, NSW 2565, Australia; 11 School of Medicine, University of Notre Dame, Darlinghurst, Sydney, NSW 2010, Australia

**Keywords:** pancreatic cancer, biomarker, neoadjuvant chemotherapy, resection, selection

## Abstract

**Simple Summary:**

Routine neoadjuvant therapy for resectable and borderline resectable pancreatic cancer is gaining popularity, but its true oncological benefit remains disputed. Whilst the genotypic and phenotypic heterogeneity of pancreatic cancer is becoming increasingly appreciated, there is currently no method to determine whether certain patients will benefit from a neoadjuvant approach and whether others will benefit from a surgery-first approach. In this study, a previously validated prognostic triple biomarker panel is shown to predict genetic subtypes and clinical phenotypes of pancreatic cancer and also the optimal treatment strategy (neoadjuvant vs. surgery-first) for patients with resectable and borderline resectable pancreatic cancer.

**Abstract:**

The genomic heterogeneity of pancreatic ductal adenocarcinoma (PDAC) is becoming increasingly appreciated. We aimed to evaluate the ability of a triple biomarker panel (S100A4, Ca-125, and mesothelin) to predict: (i) genetic PDAC subtypes; (ii) clinical phenotypes; and (iii) the optimal treatment strategy (neoadjuvant vs. surgery-first) in resectable and borderline resectable PDAC. Patients who underwent resection for resectable and borderline resectable PDAC were included from one single-institutional cohort and one multi-institutional cohort from the Australian Pancreatic Genome Initiative (APGI). Tumors were immunohistochemically evaluated for S100A4, Ca-125, and mesothelin, and a subset from the APGI cohort underwent RNA sequencing. This study included 252 and 226 patients from the single institution and the APGI cohorts, respectively. Triple-negative biomarker status correlated with non-squamous PDAC genotypes (*p* = 0.020), lower rates of distant recurrence (*p* = 0.002), and longer median overall survival (mOS) with the surgery-first approach compared with neoadjuvant treatment (33.3 vs. 22.2 mths, *p* = 0.038) in resectable PDAC. In contrast, the triple-positive disease was associated with longer mOS with neoadjuvant treatment compared with the surgery-first approach (29.5 vs. 13.7 mths, *p* = 0.021) in resectable and borderline resectable PDAC. In conclusion, the triple biomarker panel predicts genetic PDAC subtypes, clinical phenotypes, and optimal treatment strategies in resectable and borderline resectable PDAC.

## 1. Introduction

Pancreatic ductal adenocarcinoma (PDAC) continues to be associated with a dismal prognosis, with an overall 5-year survival rate of 8.7% [1,2]. Of the patients who undergo PDAC resection, up to 30% will face mortality due to disease recurrence within one year of surgery [3]. This implies that many patients endure the short- and long-term morbidity of major pancreatic resection for no survival benefit. In light of this, neoadjuvant treatment for potentially operable PDAC is fast gaining popularity and has found its utility as a “test” of tumor biology and occult micrometastases [4]. This allows clinicians to reserve morbid surgery for those patients with localized chemo-responsive diseases who do not progress on neoadjuvant therapy. Due to the lack of published randomized controlled data, this treatment approach in resectable and borderline resectable PDAC remains an area of clinical equipoise. Given the genotypic and phenotypic heterogeneity of PDAC as a disease entity, the optimal treatment strategy for patients—whether neoadjuvant or surgery-first—is likely to vary from patient to patient. Therefore, there is a need to be able to predict which treatment strategy will lead to the best survival outcome for each patient.

Recent work has demonstrated that PDAC comprises a number of genomically distinct clusters. In particular, subtypes characterized by upregulation of genes related to epithelial-to-mesenchymal transition and increased metastatic potential are associated with poor overall survival. These include the Bailey “squamous” subtype [5] and the Collisson “quasi-mesenchymal” [6] subtype. Knowledge of the genetic subtypes of PDAC allows the opportunity to explore personalized treatment pathways for patients in the future. However, the high cost of genomic sequencing renders the routine use of these genetic subtypes less viable in a clinical setting [7].

Our group has previously reported a cost-effective triple immunohistochemical biomarker panel (S100A4, Ca-125, and mesothelin) that stratifies PDAC patients into prognostically significant groups in patients undergoing upfront resection. Patients with “triple-negative” disease (all three biomarkers negative) have the longest overall survival, and patients with “triple-positive” disease are associated with the shortest median overall survival of approximately one year [8].

We hypothesized, therefore, that patients with biomarker-positive disease represent those with a “high-risk” phenotype (HR-PDAC) and a “squamous” genotype who do not derive significant survival benefits from upfront surgical resection due to a high propensity for early metastatic disease but benefit from a neoadjuvant treatment approach. On the other hand, we hypothesized that triple-negative biomarker patients represent those with a “low-risk” phenotype (LR-PDAC) and “non-squamous” genotype, those with a lesser tendency for early metastatic disease, and those who are best served with a surgery-first approach.

The aims of this study were to evaluate: (i) the relationship between triple biomarker expression and genetic PDAC subtype; (ii) the association between triple biomarker expression and clinical PDAC phenotype (LR- vs. HR-PDAC); and (iii) the association between triple biomarker expression and the degree of survival benefit gained from a neoadjuvant vs. surgery-first treatment approach in patients with resectable and borderline resectable PDAC.

## 2. Methods

### 2.1. Patient Selection

This was a cohort study of prospectively collected tissue and data. To address aims (i) and (ii), we utilized tissue and data collected as part of the multi-institutional Australian Pancreatic Genome Initiative (APGI), which was subsequently contributed to the pancreatic cancer arm of the International Cancer Genome Consortium (ICGC). To address aim (iii), local tissue and data were used from a single tertiary-level institution (Royal North Shore Hospital (RNSH)), Sydney, Australia). Consecutive patients who underwent surgical resection of resectable or borderline resectable PDAC (according to 2017 NCCN Criteria) [9] from 2010 to 2017 were included for analysis. Patients who had a locally advanced or metastatic disease or who had operative mortality were excluded from the analysis. Patients with other non-PDAC pancreatobiliary malignancies such as cholangiocarcinoma or ampullary adenocarcinoma were also excluded. The conduction of this study was approved by the Northern Sydney Local Health District Human Research and Ethics Committee.

### 2.2. Patient Treatment

For both cohorts, all patients were discussed in a multidisciplinary tumor board. In the RNSH cohort, all patients with borderline resectable PDAC were routinely offered neoadjuvant chemotherapy (NAC) prior to resection during the entire study period unless there was a clinical contraindication. NAC was prescribed at the discretion of the treating medical oncologist. From 2010 to 2016, patients with clearly resectable PDAC were offered upfront surgical resection. From the year 2016 onward, patients with clearly resectable PDAC were also routinely offered NAC prior to resection if there were no contraindications. The APGI/ICGC cohort only included patients who had undergone a surgery-first approach.

All patients underwent standard pancreatic resection. Patients were then offered adjuvant chemotherapy upon recovery from surgery. Clinical follow-up of patients was undertaken every three months for the first two years and then every six months thereafter. Follow-up chest and abdominal computed tomography (CT) scans were performed every six months for the first two years and then yearly thereafter.

### 2.3. Immunohistochemistry

Tissue microarrays (TMAs) of formalin-fixed paraffin-embedded (FFPE) PDAC specimens were formed using 1 mm tissue cores of the tumor taken from each patient in replicates of at least two and re-embedded in paraffin. Then, 4μm thick sections were taken from each TMA block, deparaffinized in xylene, rehydrated in graded ethanol, and quenched in 0.3% hydrogen peroxide. S100A4 immunostaining was performed using DAKO^TM^ (Glostrup, Denmark) anti-rabbit S100A4 primary antibody (Product A5114, 1:1000 concentration, 1 h incubation, room temperature) following heat-induced epitope retrieval (HIER) at pH 6 for 20 min. Ca-125 immunostaining was performed using DAKO^TM^ (Glostrup, Denmark) anti-mouse Ca-125 antibody (Clone M11, 1:100 dilution, 1 h incubation, room temperature) following HIER at pH 9 for 20 min. Mesothelin immunostaining was performed using Novocastra^TM^ (Newcastle upon Tyne, UK) anti-mouse mesothelin antibody (Clone 5B2, concentration 1:20, 1 h incubation, room temperature) following HIER at pH 6 for 20 min. Secondary antibody incubation was performed (EnVision^TM^ mouse/rabbit kit; DAKO, Glostrup, Denmark), followed by chromogen and then hematoxylin counterstain. Immunolabelling of all antibodies was scored by two authors (C.N., primary author; J.T., surgical pathologist), both blinded to all clinical data. Tissue cores were scored for the intensity of staining and the percentage of tumor cells stained (0 = no staining; 1 = weak staining; 2 = strong staining), as described previously [8]. The mean of the scores from both observers was taken as the final score for each patient. A mean score of >0.5 was interpreted as a positive expression, and a score of ≤0.5 was determined to be a negative expression.

### 2.4. Biomarker Combinations

Patients were categorized into groups of biomarker expression according to how many of the three biomarkers (S100A4, Ca-125, and mesothelin) were expressed. These groups have previously been demonstrated and validated as prognostically relevant [8]. Patients not expressing any of the three biomarkers were determined to be “triple-negative”. Patients expressing one, two, and three of the three biomarkers were deemed to be “single”, “double,” and “triple” positive, respectively.

### 2.5. Transcriptomic Subtypes of Pancreatic Cancer

In the APGI/ICGC cohort, 68 patients formed part of the cohort used to perform RNA sequencing (RNASeq) of tumor tissue, leading to the first description of the Bailey PDAC subtypes (pancreatic progenitor, ADEX, immunogenic, and squamous) [5]. The classification of PDAC subtypes was thus available for this group of patients.

### 2.6. Definitions of Distant and Locoregional Recurrence

Data regarding the timing and site of the first PDAC recurrence after resection were available in the APGI/ICGC cohort. “Locoregional recurrence” was defined as any histological or radiological evidence of recurrent disease in the pancreatic bed, remnant pancreas, or regional lymph nodes. Any recurrent disease not in these locations was defined as “distant recurrence” (e.g., liver, lung, peritoneum, brain, bone).

### 2.7. Data Analysis

Categorical variables were assessed for the strength and significance of association using a two-tailed Fisher’s exact test. Comparative survival analyses between groups were performed using the Kaplan–Meier method with log-rank comparison or Cox regression analysis as appropriate. Overall survival was defined as the time interval between the date of diagnosis and the date of death. Time to recurrence was defined as the time interval between the date of surgery and the date of the first radiological evidence of recurrent locoregional or metastatic disease. In addition, *p* values < 0.05 were considered statistically significant. All statistical analyses were performed using SPSS for Windows v25 (IBM Corp^TM^, Armonk, NY, USA).

## 3. Results

### 3.1. Baseline Characteristics

Baseline characteristics for both cohorts are detailed in Table 1. In this study, there were 226 patients in the APGI/ICGC cohort and 252 patients in the RNSH cohort.

### 3.2. Biomarker Panel Association with Propensity for Early Disease Recurrence

In the APGI/ICGC cohort (*n* = 226), double-positive (HR 1.654, *p* = 0.048) and triple- positive biomarker status (HR 2.136, *p* = 0.002) was characterized by higher rates of distant disease recurrence compared with triple-negative biomarker status (Table 2). Thus, double- and triple-positive biomarker disease was associated with HR-PDAC, and triple-negative disease was associated with LR-PDAC. A greater hazard of locoregional PDAC recurrence was seen with single- (HR 1.893, *p* = 0.036) and double-positive disease (HR 2.536, *p* = 0.005) compared with triple-negative disease.

### 3.3. Biomarker Panel Association with Non-Squamous Subtype

Table 3 demonstrates the relationship between biomarker status and transcriptomic subtype of PDAC. In the APGI/ICGC cohort with available PDAC subtype data (*n* = 68), a “triple-negative” pattern of biomarker expression was significantly associated with the non-squamous subtype of PDAC (OR 5.804, 95% CI 1.191–28.271, *p* = 0.020). S100A4 (OR 2.475, 95% CI 0.766–7.997, *p* = 0.148) and Ca-125 (OR 2.684, 95% CI 0.828–8.698, *p* = 0.137) expression individually demonstrated a non-significant trend toward association with the squamous PDAC subtype. Single-positive (OR 3.341, 95% CI 0.994–11.229, *p* = 0.093), double-positive (OR 2.848, 95% CI 0.685–11.847, *p* = 0.210), and triple-positive disease (OR 0.587, 95% CI 0.115–2.998, *p* = 0.717), when considered separately, were not significantly associated with the squamous subtype. However, when these biomarker patterns were considered as a group (i.e., single + double + triple positive), there was a significant association with the squamous subtype (OR 5.804, 95% CI 1.191–28.271, *p* = 0.020).

### 3.4. Biomarker Correlation with Neoadjuvant Treatment Outcomes

**Figure 1:**
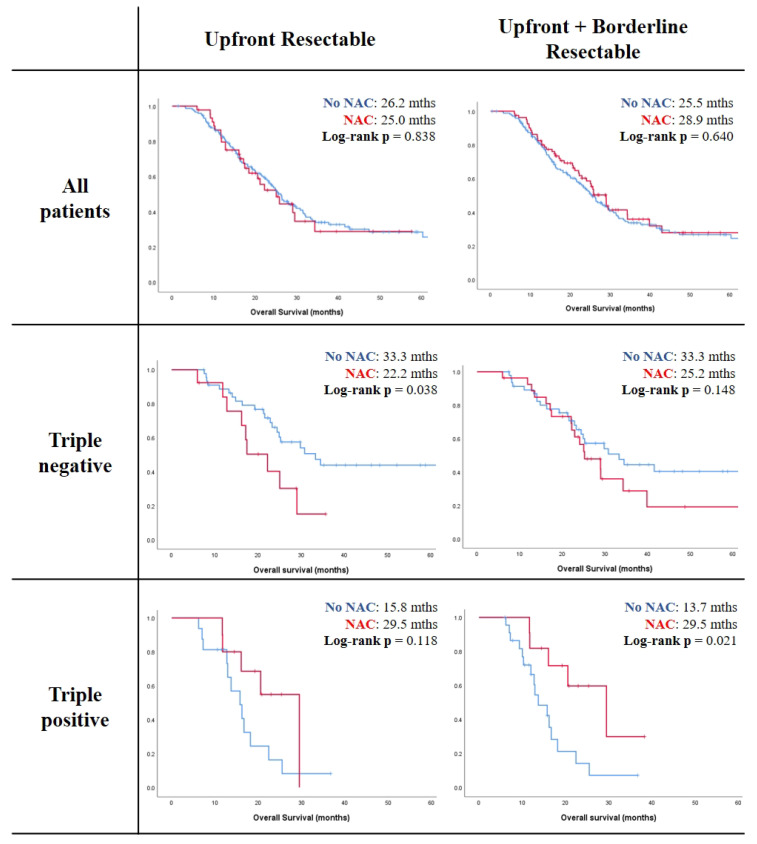
Influence of triple biomarker expression on survival outcomes after a neoadjuvant vs. surgery-first treatment strategy in the RNSH cohort: NAC, neoadjuvant chemotherapy; resectable PDAC cohort, *n* = 192; resectable + borderline resectable cohort, *n* = 252.

In the RNSH cohort (*n* = 252) of resectable and borderline resectable patients, there was no significant difference in overall survival between patients who received NAC and those who underwent upfront resection (Figure 1). However, subgroup analysis revealed that in patients with triple-positive biomarker status (HR-PDAC), NAC was associated with significantly longer overall survival compared with upfront resection (29.5 vs. 13.7 months, log-rank *p* = 0.021). In contrast, in upfront resectable patients with triple-negative biomarker status (LR-PDAC), NAC was associated with significantly shorter overall survival compared with patients who underwent upfront resection (22.2 vs. 33.3 months, log-rank *p* = 0.038). No significant difference was seen in overall survival between NAC and upfront resected groups in both single- and double-positive patients (log-rank *p*-value > 0.2). Therefore, all three biomarkers are needed to identify the patients who will benefit from NAC.

## 4. Discussion

**Figure 2:**
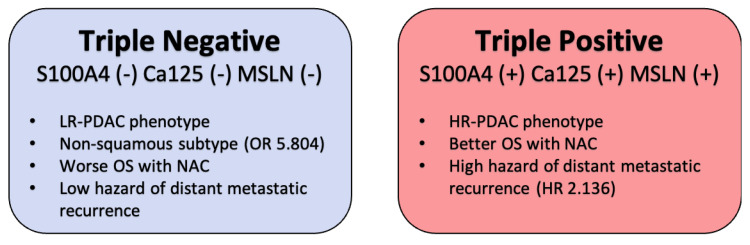
Summary of findings: LR-PDAC, low-risk pancreatic ductal adenocarcinoma; HR-PDAC, high-risk pancreatic ductal adenocarcinoma; OS, overall survival; NAC, neoadjuvant chemotherapy.

In this study, we demonstrated for the first time that triple-positive biomarker status is associated with an HR-PDAC phenotype with a higher risk of early metastatic disease and is best served with neoadjuvant treatment, followed by resection. It is also reported that triple-negative biomarker status is associated with the less aggressive non-squamous PDAC genotypes and the LR-PDAC phenotype with a lower risk of early metastatic disease, and it is best served with a surgery-first approach rather than neoadjuvant treatment (Figure 2).

The heterogeneity of the genomic landscape and clinical behavior of PDAC is becoming increasingly appreciated [5,6,10,11]. In addition to this, the added influences of epigenetic modification, tumor–stromal interaction, and host immune response translate to great variability in clinical phenotypes, particularly with regard to the propensity for early metastatic recurrence and the benefits of neoadjuvant treatment. Yet, current pre-operative staging criteria [12], which are based heavily on imaging modalities, give only a snapshot of the local and/or distant status of the tumor and very little account for its biological behavior.

Currently, there is increasing use of neoadjuvant treatment strategies [13] as a test of tumor biology [4], allowing occult metastatic disease an opportunity to declare itself in an effort to avoid morbid surgery in patients unlikely to benefit from major resection. However, the lack of randomized controlled data for the use of this treatment strategy in resectable patients means that it cannot be routinely recommended, despite its increasing popularity. The findings of the present study raise concerns that some patients, particularly those with an LR-PDAC phenotype, would experience inferior survival outcomes with routine neoadjuvant treatment compared with a surgery-first approach. The NCCN guidelines still stipulate that upfront resection is the standard of care and that neoadjuvant therapy may be considered in “high-risk” patients [12]. This consequently remains an area of debate and clinical equipoise. To date, the problem has remained, however, in identifying patients who are in this “high-risk” category.

A number of studies of gene expression in PDAC have been able to identify a “high-risk” cluster characterized by upregulation in gene programs associated with invasion and metastasis, greater propensity for metastatic recurrence, and poor overall survival. These include the Bailey “squamous” [5], the Moffitt “basal-like” [11], and the Collisson “quasi-mesenchymal” [6] subtype. However, despite significant advances in DNA sequencing, this remains unfeasible for routine clinical use due to economical and logistic barriers. Based on a recent cost-modeling analysis, whole-genome sequencing, whole-exome sequencing and targeted gene panels were EUR 1669 (USD 1895), EUR 792 (USD 899), and EUR 333 (USD 378), respectively [7]. Logistic barriers associated with DNA sequencing were highlighted by the IMPaCT trial, in which genetic analysis data could not be returned in a timely fashion to up to 25% of study participants [14].

In contrast, immunohistochemical biomarker profiling currently provides a clinically useful and economically viable alternative for identifying the high-risk PDAC patient at approximately USD 60 per patient for three biomarkers [1]. As immunohistochemistry is a routinely performed technique in hospital anatomical pathology laboratories, there is no requirement for additional infrastructure, training, or personnel. From the time of specimen retrieval, immunohistochemical analysis can be performed within 48 h, allowing for formalin fixation, paraffin embedding, and IHC analysis. Furthermore, immunohistochemistry reflects the sum of epigenetic modifications that occur downstream of the genome, thus providing a more accurate depiction of the disease phenotype [15].

In our previous work, the triple biomarker panel comprising S100A4, Ca-125, and mesothelin was able to stratify patients with upfront resected PDAC into groups with distinct overall survival outcomes [8]. These findings were validated in an externally derived cohort. In particular, the high-risk HR-PDAC group with “triple-positive” disease had an overall survival of 11.9–12.8 months after resection. In contrast, the “triple-negative” low-risk LR-PDAC group of patients had the most favorable prognostic outcome, with a median overall survival of up to 36.8 months. The biological explanation for this is based on the roles played by S100A4, Ca-125, and mesothelin in promoting tumor invasion and metastasis [8,16,17,18]. The expression of each additional one of these three biomarkers contributes to an increasingly HR-PDAC phenotype and the likelihood of occult micrometastatic disease. On the other hand, the expression of none of these biomarkers (triple-negative) represents an LR-PDAC phenotype with a lower propensity for micrometastatic disease.

The present work has demonstrated that in patients with a high-risk HR-PDAC phenotype (triple-positive), neoadjuvant treatment is associated with longer overall survival. In contrast, in patients with a low-risk LR-PDAC phenotype (triple-negative), neoadjuvant treatment was associated with significantly worse overall survival. This may be due to a delay of a much-needed resection of the low-risk LR-PDAC primary tumor, and it may reflect a lost opportunity to remove the culprit lesion prior to the development of metastases. In support of this, it has previously been demonstrated that a delay in the interval from radiological diagnosis to surgery leads to an increased rate of unexpected metastatic disease [19,20].

There is, therefore, a strong case for routine biomarker profiling in potentially resectable PDAC patients to match patient phenotypes to an appropriate treatment strategy. The present study utilized 1 mm circular cores of tumor tissue to analyze immunohistochemical biomarker status, giving a core area of approximately 3.14 mm^2^. This is less than the area provided by an endoscopic core biopsy of pancreatic lesions, which provides 3.43 mm^2^ of tissue area based on assumptions of a 19-gauge core needle (internal diameter 0.686 mm) and a core length of 5 mm. This strongly suggests the feasibility of preoperative assessment of biomarker status on core biopsy samples of pancreatic tumors, which can be obtained with negligible complication rates [21]. Immunohistochemical assessment of S100A4, Ca-125, and mesothelin has also been demonstrated as feasible using fine needle aspirates of PDAC tumors [22,23,24].

The present study showed that triple-negative biomarker status (LR-PDAC) was associated significantly with the non-squamous genotype. Only S100A4 positivity was associated with a trend toward the squamous genotype, but we were unable to show statistical significance as reported by Dreyer et al. [22]. This is likely due to subtle variations in the antibody and methodology used for immunohistochemical staining, and a lower number of available patients (*n* = 68) with paired immunohistochemical and RNA sequencing data in the present study compared with Dreyer et al. (*n* = 235). However, the expression of at least one of three biomarkers in the current study was significantly correlated with the squamous subtype. These findings are also in alignment with our previous work which demonstrated close clustering of three biomarkers (i.e., S100A4, Ca-125, and mesothelin) with the markers for squamous PDAC subtype [25].

The present study has some limitations. Biomarker evaluation was performed on cores derived from paraffin blocks of resected PDAC specimens for this study but should be prospectively evaluated in the future on endoscopically derived core biopsy specimens. Additionally, transcriptomic subtype data were available for only a small proportion of the patients in the APGI cohort.

## 5. Conclusions

Triple-negative biomarker status (LR-PDAC phenotype) is associated with non-squamous subtypes of pancreatic cancer, and it is associated with worse survival outcomes if resection is delayed due to neoadjuvant treatment. In contrast, triple-positive biomarker status (HR-PDAC) is associated with better survival outcomes with neoadjuvant treatment prior to resection. Future prospective trials are required to investigate the feasibility of using the triple biomarker panel on endoscopic core biopsy specimens to help guide treatment for PDAC patients.

## Figures and Tables

**Table 1:** Baseline characteristics of the ICGC and the RNSH cohort.

Characteristic	ICGC (*n* = 226) Number (%) Median (Range)	RNSH (*n* = 252) Number (%) Median (Range)	*p*-Value
**Age**	67 (34–88)	68 (33–86)	0.482
**Gender, male**	113 (50.0)	131 (51.9)	0.714
**Follow-up, months**	21.2 (0.03–99.4)	22.0 (0.4–105.6)	0.608
**Overall survival, months**	21.4	25.8	0.076
**Adjuvant chemotherapy**	162/181 (89.5)	144/184 (78.3)	0.004
Tumor **size, mm**	34 (10–90)	30 (3–100)	0.169
**T-stage**			
**1–2**	16/225 (7.11)	14/225 (6.22)	0.572
**3–4**	209/225 (92.9)	238/252 (94.4)	
**Lymph node** positive	166/224 (74.1)	183/252 (72.6)	0.756
Lymphovascular invasion	135/219 (61.6)	143/251 (56.9)	0.347
Perineural invasion	182/222 (81.9)	177/250 (70.8)	0.005
**R1 resection**	65/225 (28.9)	112/252 (44.4)	<0.001
**Biomarker pattern**			
**S100A4 positive**	186/224 (83.0)	164/241 (68.0)	<0.001
Ca-125 **positive**	114/224 (50.9)	133/242 (54.9)	0.404
**MSLN positive**	126/224 (56.3)	112/245 (45.7)	0.026
**Triple negative**	21/223 (9.42)	38/236 (16.1)	0.009
**Single positive**	63/223 (28.3)	59/236 (25.0)	
**Double positive**	54/223 (24.2)	76/236 (32.2)	
**Triple positive**	85/223 (38.1)	63/236 (26.7)	

**Table 2:** Biomarker panel and hazard of distant and locoregional recurrence in the APGI/ICGC cohort.

Distant Recurrence			
Biomarker Pattern	β Coefficient	Hazard Ratio (95%CI)	*p*-Value
Triple negative	Reference	Reference	Reference
Single positive	0.294	1.342 (0.855–2.105)	0.201
Double positive	0.503	1.654 (1.004–2.723)	0.048
Triple positive	0.759	2.136 (1.317–3.464)	0.002
**Locoregional Recurrence**			
**Biomarker Pattern**	**β Coefficient**	**Hazard Ratio (95%CI)**	***p*-Value**
Triple negative	Reference	Reference	Reference
Single positive	0.638	1.893 (1.042–3.441)	0.036
Double positive	0.930	2.536 (1.326–4.848)	0.005
Triple positive	0.465	1.592 (0.738–3.438)	0.236

**Table 3:** Patterns of biomarker expression compared with transcriptomic subtypes of pancreatic cancer.

	PP (*n* = 22)	ADEX (*n* = 10)	IG (*n* = 21)	Squamous (*n* = 15)	Non-Squamous (*n* = 53)	OR (Squamous vs. Non-Squamous)	*p*-Value (Squamous vs. Non-squamous)
**S100A4 positive (*n* = 51)**	17	7	13	14	37	2.475	0.148
**Ca-125 positive** **(*n* = 33)**	12	5	7	9	24	2.684	0.137
**MSLN positive (*n* = 34)**	12	6	10	6	28	0.595	0.560
**Triple negative (*n* = 8)**	2	2	4	0	8	0.172	0.020
**Single positive (*n* = 24)**	8	2	8	6	18	3.341	0.093
**Double positive (*n* = 14)**	3	2	5	4	10	2.848	0.210
**Triple positive (*n* = 22)**	9	4	4	5	17	0.587	0.717

PP, pancreatic progenitor; ADEX, aberrantly differentiated endocrine exocrine; IG, immunogenic; MSLN, mesothelin; OR, odds ratio.

## Data Availability

The data that support the findings of this study are available from the corresponding author upon reasonable request.
